# Size Selection of Antarctic Krill (*Euphausia superba*) in Trawls

**DOI:** 10.1371/journal.pone.0102168

**Published:** 2014-08-08

**Authors:** Ludvig A. Krag, Bent Herrmann, Svein A. Iversen, Arill Engås, Sigve Nordrum, Bjørn A. Krafft

**Affiliations:** 1 DTU Aqua, Technical University of Denmark, Hirtshals, Denmark; 2 SINTEF Fisheries and Aquaculture, Fishing Gear Technology, Hirtshals, Denmark; 3 Institute of Marine Research, Bergen, Norway; 4 Aker BioMarine Antarctic AS, Oslo, Norway; Aristotle University of Thessaloniki, Greece

## Abstract

Trawlers involved in the Antarctic krill (*Euphausia superba*) fishery use different trawl designs, and very little is known about the size selectivity of the various gears. Size selectivity quantifies a given trawl's ability to catch different sizes of a harvested entity, and this information is crucial for the management of a sustainable fishery. We established a morphological description of krill and used it in a mathematical model (FISHSELECT) to predict the selective potential of diamond meshes measuring 5–40 mm with mesh opening angles (oa) ranging from 10 to 90°. We expected the majority of krill to encounter the trawl netting in random orientations due to high towing speeds and the assumed swimming capabilities of krill. However, our results indicated that size selectivity of krill is a well-defined process in which individuals encounter meshes at an optimal orientation for escapement. The simulation-based results were supported by data from experimental trawl hauls and underwater video images of the mesh geometry during fishing. Herein we present predictions for the size selectivity of a range of netting configurations relevant to the krill fishery. The methods developed and results described are important tools for selecting optimal trawl designs for krill fishing.

## Introduction

The largest annual catch of Antarctic krill (*Euphausia superba*, hereafter called krill) in the Southern Ocean since the inception of a commercial fishery in 1972 was 528,000 tons. During the last decade, the annual catches in the Scotia Sea, where the commercial operations are located today, have ranged between 100,000 and 212,000 tons. Due to limited knowledge about this marine ecosystem and the potential negative effects of fishery activities, a precautionary catch limit for the Scotia Sea was set at 620,000 tons by the Commission of Antarctic Marine Living Resources (CCAMLR) in 1991 to avoid potential conflicts with land-based predators that depend on krill as prey such as penguins [1]. Based on a coordinated survey conducted by the CCAMLR in 2000, during which krill density was measured acoustically in the fishing areas [2], the biomass of krill was calculated to be 60.3 million tons [3]. A theoretical total allowable catch (TAC) limit based on this calculation was set at 5.61 million tons [3].

The pelagic trawlers involved in the krill industry use different trawl systems and designs, and very little is known about the size selectivity of the commercial trawls used to harvest krill. Pshenikov [4] reported that during the 1970s when the Soviets trawled for krill, only 10–20% of the krill that entered the trawl opening were retained in the codend. However, neither data nor analyses were provided in this paper. In the near future, the krill harvest is expected to reach the precautionary catch limit due to increased demand for krill and improved harvesting and processing technologies. The potential to increase the annual catch further (i.e., to the TAC limit in the Scotia Sea) is significant [3], [5]. Krill have a circumpolar distribution [6], which will enable a spatial expansion of the fishery that potentially could contribute to increases in the total annual catch. The objectives of responsible harvesting of krill, rational management of the krill fishery, and economic profit for the industry demand development of fishing gear that reduces accidental (escape) mortality during the fishing process. Both the Commission and Scientific Committee of the CCAMLR strongly recommend member states that are fishing for krill to investigate the effects of different fishing gears on krill escapement to assess the total mortality of the krill stock caused by the fishery [7], [8].

Studies of size selectivity for other commercially harvested species in towed fishing gear have traditionally been made by conducting a series of sea trails. Sea trials are economically costly and time consuming, and there is therefore a limit to the number of different gear designs that can be tested. In addition, some commercial krill fishing vessels use a continuous pumping technique to bring the catch from the trawl to the deck, which makes standard selectivity studies difficult. Due to these limitations, the FISHSELECT method was used in the present study to evaluate size selectivity of relevant gears for the krill fishery [9]. FISHSELECT is based on a combination of laboratory experiments and computer simulations which involves assessment of the morphology relevant for size selection. This method has been used successfully to describe, understand, and predict size selection of fish [9], [10], [11], [12], [13] and crustaceans, such as *Nephrops norvegicus* [14]. The essential first step of the method includes collecting morphometric measurements of the species investigated and estimating selectivity parameters based on comparing the morphology of the species and the geometry of relevant meshes. This procedure makes it possible to quantify the size selectivity of different trawl designs, including those commercially used today. Moreover, it constitutes an essential step towards evaluating escape mortality in the trawl-based krill fishery. The ability to quantify and predict size selectivity in the commercial fishery for krill will allow managers to explore the consequences of fishing in terms of catch efficiency and catch loss of different gear designs when harvesting populations with dissimilar size structures.

In general, fish targeted by trawls have good swimming ability relative to the towing speed used in these fisheries. Several species of fish also have been observed to orientate themselves in relation to the trawl netting during the capturing process e.g., [15]. In contrast to fish, smaller invertebrates such as prawns tend to display a more limited response to stimuli from the trawl [16], [17]. Krill are similar in size to the smaller species of commercially fished prawns, but they are fished using towing speeds similar to those used for targeting fish (i.e., 2.5–3.0 knots). Therefore, the selectivity process for krill in trawls is expected to resemble a sieving process in which the individual krill may meet the trawl netting with a more random orientation (in contrast to what has been observed for fish). Trawls designed to catch small crustaceans like krill or shrimps are designed with small meshes in the entire trawl indicating similar expectations from the fishermen.

The objective of this study was to establish a morphology based model for krill to predict size selectivity of a range of netting configurations relevant to the krill fishery. Simulation-based results (FISHSELECT, [9]) were compared with results from a selectivity experiment that involved hauling two different trawl gear design, a macroplankton survey trawl and commercial trawl simultaneously through a krill swarm. To explore behavioral patterns of krill during fishing, underwater video observations were made.

## Materials and Methods

### Ethics statement

This study did not involve endangered or protected species. Experimental fishing was conducted onboard a Norwegian commercial trawler. No permit was required to conduct the study.

### Morphological description of krill

We used the FISHSELECT method with some modifications to establish a morphological description of krill. The FISHSELECT method aims to identify and parameterize the morphological properties of a given species that determine the species size selection of towed fishing gear. For some fish species the vital morphology has been identified as one or two cross sections along the length axis that contains the maximum compressed height and/or the maximum width due to the cylindrical shape of fish such as gadoids [9], [10]. Descriptions of these cross sections are then used to predict the size selectivity by examining whether or not the cross section is able to pass through the mesh geometry, which can be of a variety of sizes and shapes. Traditionally, the FISHSELECT method includes a series of “fall-through trials” [9] to estimate the compressibility of the identified cross sections for fish, as fish are flexible to a certain point and can compress their cross section shape during mesh penetration. However, crustaceans have a hard exoskeleton, so this procedure is not necessary for krill [14]. To date, the FISHSELECT methodology and the measuring tools have not been applied to crustaceans as small as krill, which can grow to a maximum total body length of 60 mm (e.g., [18]). Thus, application of the FISHSELECT method to krill requires further development of some of the procedures. The major steps involved in the method are described in detail in Herrmann et al. [9], and the customization for using the method on krill is elaborated below.

### Collecting krill and studying the effect of preservation

Fresh krill were not available for the experimental laboratory trials needed for this study, so preserved animals were used in these trials. Determining if and how the preservation treatment changed the morphology of krill were important for accurate interpretation of the results. When Germany first initiated its krill research program in the Southern Ocean in 1976, researchers compared fresh, frozen, ethanol-preserved, and formalin-preserved krill samples to evaluate potential effects of preservation on morphological properties. They observed shrinkage in samples preserved in ethanol, but no statistically significant differences were detected in formalin fixed or frozen animals (Volker Siegel, pers comm.). Because these factors are fundamental for the reliability of our results, and because these historical results never were published, this result required corroboration.

During a survey conducted aboard the Norwegian fishing vessel Juvel (Emerald Fisheries ASA) off the South Orkney Islands in late January to early February 2012, krill were collected fresh from the catch using a Macroplankton trawl (see [18] for additional descriptions of the trawl). The trawl was lowered from the sea surface to 200 m depth and hauled at 2.5–3 knots. Sex and maturity stages of krill were determined using the classification methods outlined by [19]. Total body length was measured (±1 mm) from the anterior margin of the eye to the tip of telson, excluding the setae, according to the “Discovery method” used in [20]. Carapace width was measured using a caliper at its widest cross section point. A total of 30 krill including juveniles, sub adults, adults with gravid females at stage FIIID were preserved individually in borax-buffered formalin (4%), and body length and carapace width were measured again after 2 and 10 months.

Comparisons of any temporal change in the morphology measurements was made using an analysis of variance test (Proc NPAR1WAY, SAS Institute Inc., Box 8000, Cary, N.C., U.S.A.) with the 0.05 level accepted as indicating statistical significance.

### Morphological measurements

**Figure 1:**
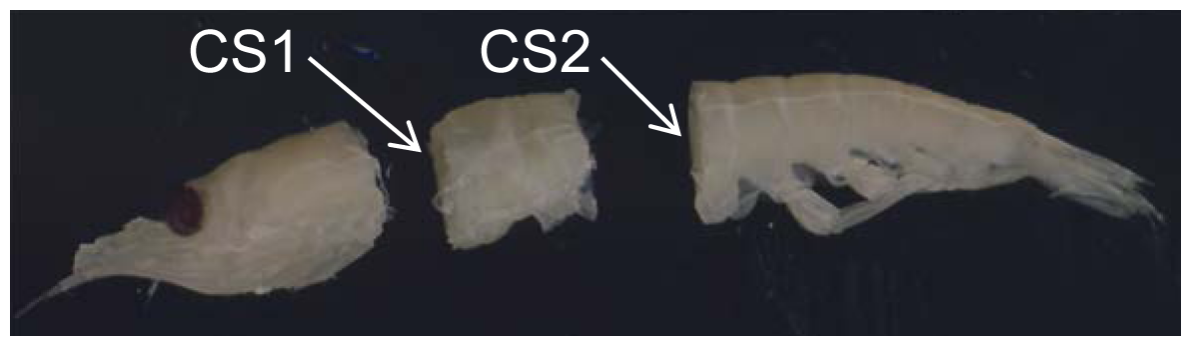
Krill sliced at cross section 1 (CS1) and cross section 2 (CS2).

Before initiation of the laboratory trials, the krill body was examined to identify the relevant morphology that potentially would determine the animal's ability to penetrate different meshes. We first determined the body orientation that would allow the largest individual to escape through a given mesh, as this orientation is indicative of the selective potential for krill for that mesh type. The optimal orientation for krill is when the body is stretched and meets the mesh opening head or tail first. We next identified morphological measures that could describe this optimal orientation. Two cross sections, CS1 and CS2, were identified along the length axis of the krill body as being decisive for size selection, as these cross sections contain the maximum height (h) and width (b) measures of animals in the optimal orientation (Fig. 1).

**Figure 2:**
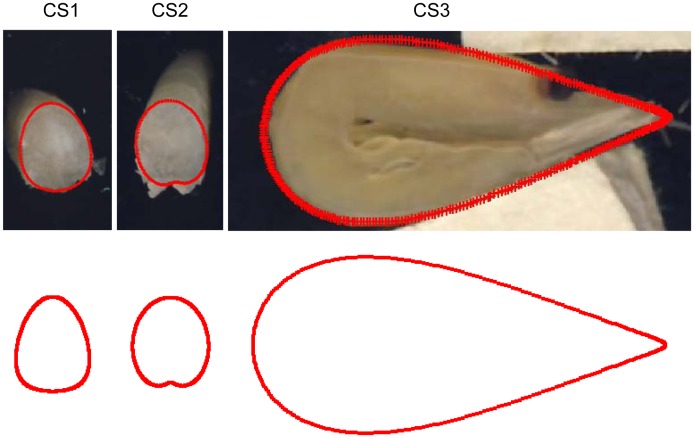
Shapes of CS1, CS2 and CS3. CS1 is described by a flexellipse_1, CS2 is described by a flexellipse_3 and CS3 is described by a flexdrope_2 (see appendix S1).

We also considered the different ways in which krill can meet the meshes in a trawl (i.e., head first, tail first, curled, stretched), as this determines which additional measurements should be made during data collection. In this study we included a second contact mode, the curled shape, CS3 (Fig. 2). We expect that krill are more passive in a trawl compared with fish and thus might encounter the meshes in more random orientations. CS3 represents a contact mode very different from the optimal mode, and it is expected to result in low selectivity due to the large cross section shape. At this stage we chose to include only one contact mode in addition to the optimal contact mode. If the observed size selectivity of krill is difficult to explain by simulation the process using the optimal contact mode is a more detailed approach, involving more contact modes needed.

**Figure 3:**
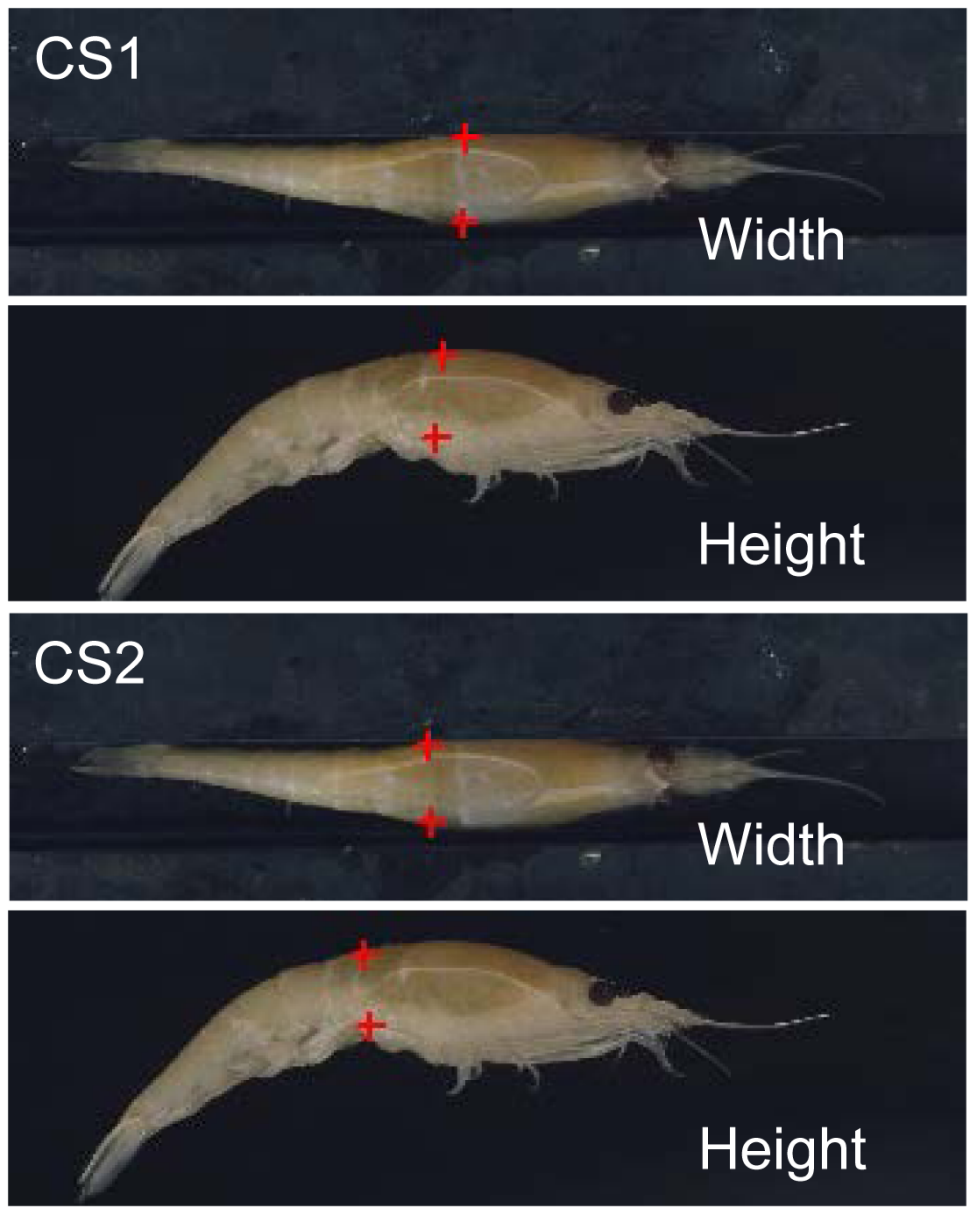
Scanned pictures of krill with markings to illustrate the width and height at CS1 and CS2.

A two-step process was used to take morphological measurements. The first step involved taking measurements of 30 individuals covering the length span from 27 to 55 mm. Each individual was placed on a flatbed scanner to measure width and height at CS1 and CS2. Width and height were extracted using the image analysis in the FISHSELECT software (Fig. 3). After these measurements were taken, each individual was frozen on a metal plate at −80°C to ensure rapid freezing. The frozen individuals were cut with a scalpel at CS1 and CS2 perpendicular to the length of the body. The frozen condition prevented the specimens from deforming during the slicing, which would cause deviation from their natural morphology. These sliced cross sections were photographed on a flatbed scanner. Then based on the shape describtion by analyzing the cross section images, we found relations between the three parameters describing the cross section shapes and size and the height and width at the cross sections. This enabled us to assess the cross section shapes and sizes established for CS1 and CS2 for a larger number of individuals based on only measuring height and width at CS1 and CS2. In the second step, the length of 83 individuals with a length range from 19 to 54 mm was measured and the established length to cross-section relationship applied. Without cutting we assessed the cross sections CS1, CS2, and CS3 by placing a given specimen on the scanner for the different measures (height, width, and curled).

### Modeling of cross sections

To describe the shapes of the the cut cross sections of CS1 and CS2 (fig 2), the best parametric model was selected for each based on R^2^ and Akaike's Information Criterion (AIC) [21]. Ten different geometric models were tested for both CS1 and CS2 (see Appendix S1). Models that fit the data well have a high R^2^ value. Comparison between models with different numbers of parameters can be made using AIC values following the procedure described in Sistiaga et al. [11], and the model with the lowest AIC value is the one that best fits the data. A more detailed mathematical description of the chosen models is given in the Appendix S1. Each model is defined by three parameters (c1, c2, and c3). For each cross section (CS1 and CS2), we have a dataset consisting of measurements from the 30 individuals that includes the values for b, h, c1, c2, c3 and a cross section model. To be able to estimate a cross section of a krill specimen based on measurements of *b* and *h* at that cross section requires knowing how c1, c2, and c3 depend on b and h for the selected models. Therefore, for each cross section we assumed that cx (x = {1,2,3} can be modeled by a simple bilinear function in b and h including interaction. Thus we use:
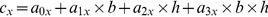
(1)Model (1) was applied using the lm function in statistical package R (version 2.15.2; www.r-project.org) to establish the models for c1, c2, and c3 for both CS1 and CS2. Each model term in (1) that was found to be non-significant was removed to establish the final models for how c1, c2, and c3 can be estimated based on b and h measured for each cross section (CS1 and CS2). The resulting models (Table 1) were subsequently applied to estimate the cross section shapes of other krill individuals based on measuring only b and h at CS1 and CS2.

**Table 1:** Model parameters and fit-statistics for model enabling that descriptions of CS1 and CS2 can be created based on height and width values of the cross sections alone.

		CS1			CS2		
		*c_1_*	*c_2_*	*c_3_*	*c_1_*	*c_2_*	*c_3_*
Model		*a_1_×b*	*a_1_×b+a_2_×h*	*a_2_×h+a_3_×b×h*	*a_1_×b+a_2_×h*	*a_1_×b+a_2_×h*	*a_2_×h+a_3_×b×h*
DF		29	28	28	28	28	28
R^2^		0.9892	0.9887	0.6831	0.9929	0.9902	0.8713
a0	Value	*	*	*	*	*	*
	SE	*	*	*	*	*	*
	p-value	*	*	*	*	*	*
a1	Value	0.5224	0.4403	*	0.2768	0.3456	*
	SE	0.0010	0.1027	*	0.0644	0.1000	*
	p-value	<2e-16	1.9e-4	*	1.9e-4	1.8e-3	*
a2	Value	*	0.2069	0.1584	0.1473	0.2126	0.1247
	SE	*	0.0932	0.0423	0.0566	0.0878	0.0267
	p-value	*	3.5e-2	8.2e-4	1.5e-2	2.2e-2	4.7e-5
a3	Value	*	*	-0.0190	*	*	-0.0129
	SE	*	*	0.0082	*	*	0.0056
	p-value	*	*	2.8e-2	*	*	2.8e-2

*not in model

### Assessment of mesh shape and size of trawl netting

**Figure 4:**
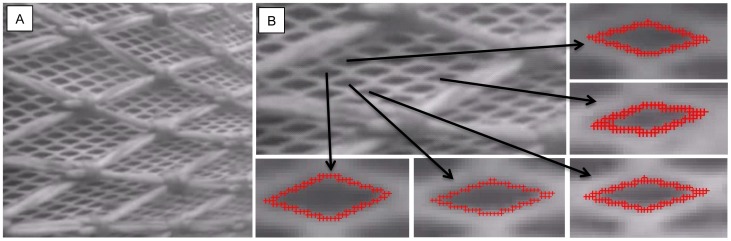
Image of the netting wall (mesh size15.4 mm) of the krill trawl captured during fishing operations (A). Digitizition of selected meshes to establish realistic values for mesh opening angles (oa) (B). The camera is located 10 m from the codline-end, pointing backwards. The intire 15.4 mm trawl was covered with 200 mm double 5 mm PE diamond netting for protection.

In addition to a morphological description of krill, we needed a precise description of sizes and shapes of the meshes used in commercial trawls for which experimental size selectivity data were collected. Weaved diamond mesh polyamide (PA) netting with a nominal mesh size of 16 mm (stretched inside measure) is commonly used in the commercial krill fishery. A small sample of this netting was placed on a flatbed scanner with no tension in the netting together with a measuring unit to determine the precise mesh size. Individual meshes in the picture were analyzed in FISHSELECT using the built-in image analysis function, and mesh size was assessed following the procedures described in Sistiaga et al. [11]. Standard mesh measuring methods (e.g., the OMEGA measuring gauge [22]), which are applied for larger mesh sizes, could not be used in this study because the measuring jaws are too large for the small mesh sizes used in the krill fishery. We used underwater video recordings made during commercial fishing operations onboard the Norwegian vessel Antarctic Sea during the 2013 season to assess the shapes of the meshes during fishing. Following Sistiaga et al. [11], the digitized images were used to extract the mesh opening angle (oa) to identify the best shape description of the meshes (e.g., diamond, hexagonal, square) (Fig. 4).

### Predicting potential size selectivity of krill

We generated a combined model of CS1 and CS2 because together they contained the maximum cross section width (CS1) and height (CS2). CS3 represents a different body shape mode by which krill can make contact with the trawl netting during fishing, so it was not combined with the other cross sections and instead was treated separately. Using the established cross section descriptions (CS1, CS2, and CS3) we conducted simulations to predict the basic selective properties for a variety of different mesh sizes and shapes using the optimal orientation of CS1_CS2 and CS3. Optimal orientation includes optimal rotation of the cross section description in the given mesh using a 90° attack angle relative to the mesh opening. The optimal orientation therefore gives the absolute potential and the upper limit for the potential size selection of the mesh sizes and shapes investigated. The standard FISHSELECT predictions of size selectivity are based on optimal orientation of the identified cross section descriptions. Such predictions of size selectivity are based only on the species morphology and on the given mesh shape and size and do not take into account any behavioral effects that may affect size selectivity of the species in question.

Herein we made selective predictions over a relevant range of diamond meshes with varying sizes and opening angles. To test a sufficient number of individuals in the selective range of all relevant mesh sizes, we created a large virtual population. The empirically established relationship between body length and the cross section shape parameters (c1, c2, c3) was used to define the properties of the individuals in the virtual population. We estimated L50 by assuming a logistic selection curve and treated the simulated penetration data as covered codend data [23]. L50 is the length at which there is 50% retention likelihood for the individual [23], and we used this and the selection range (SR).These basic selective properties were assessed for the relevant mesh sizes and shapes for each mesh type, and they are presented as iso-L50 plots known as design guides (see [9] for further details about this procedure).

### Selective effect of cross section orientation and attack angle

In previous studies carried out using FISHSELECT to predict size selectivity for different species of fish, it was assumed that each individual is optimally orientated when attempting to pass through the meshes. However, we expect krill to have a lower probability of meeting the meshes in the optimal orientation with optimal attack angle due to the relatively high towing speed compared to the size of the animal. In addition, the tapering of commercial krill trawls differs from that of traditional fish trawls, and this can affect how the individuals in the trawl meet the mesh. The commercial trawl used to collect the experimental data had a mouth opening of about 20×20 m, and the trawl was about 200 m long. This small amount of tapering resulted in an attack angle of less than 3° between the direction of the flow and the netting wall.

**Figure 5:**
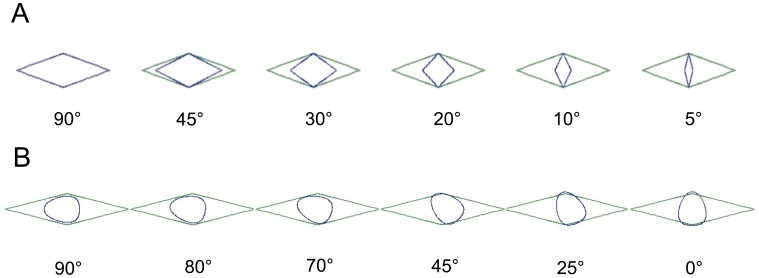
Illustration of how the effective mesh openings in a trawl can vary depending of the attack angle of krill (5–90°) (A). The light mesh is the real mesh in 90° view and the dark mesh is the effective mesh opening when projecting the mesh opening to a plan perpendicular to the towing direction. The lower mesh series (B) shows the potential effect of rotation of the cross section (CS1_CS2) in a 30° open mesh.

We explored the potential effect of orientation of CS1_CS2 in the mesh opening and the effect of attack angles between krill and the netting for the specific gear for which we collected experimental size selection data. In principle, orientation of the cross section and the attack angle work in combination, but for simplicity we assessed their potential effects separately. We therefore examined the effect of orientation of CS1_CS2 using an optimal attack angle; in this scenario, the description of CS1_CS2 was presented perpendicular to the mesh (90° in Fig. 5 Panel A) and then was rotated stepwise from 0 to 90° (Fig. 5 Panel B). To evaluate the effect of the attack angle was the mesh shape stepwise projected to a plan perpendicular to the towing direction in steps of 10° (Fig. 5). The description of CS1_CS2 during this procedure was orientated optimally in the projected mesh shape. This analysis was conducted using the FISHSELECT software.

### Collecting experimental selectivity data

Experimental size selectivity data were collected in February 2011 onboard the Norwegian commercial krill trawler Saga Sea (Aker Biomarine ASA) off the South Orkney Islands. The Saga Sea was equipped with a twin trawl beam system. One beam was rigged with a 7 mm macroplankton trawl with a 38 m^2^ mouth opening. The other beam was rigged with a commercial trawl with a 400 m^2^ mouth opening and a mesh size of 15.4 mm from the trawl opening to the rear end. The trawls were lowered from the sea surface to 200 m depth simultaneously and then slowly hauled at a vessel speed of 2.5–3.0 knots, which corresponds to the towing speed used during commercial fishing. At the surface, one trawl was taken onboard before the other trawl. The order in which each trawl that was taken onboard first was alternated between a total of four hauls. When a trawl catch was landed on deck, the body length of about 100 individual krill from each trawl was measured (±1 mm) from the anterior margin of the eye to the tip of telson, excluding the setae. A total of 416 individuals were measured from the macroplankton trawl and 393 individuals from the commercial 15.4 mm trawl.

### Analyzing experimental selectivity data

The experimentally collected data were analyzed using SELNET [12] and paired gear analysis [23]. Data were modeled by the traditional logistic model (2) with parameters L50 and SR. 
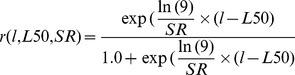
(2)The following function (3) was minimized with respect to the parameters L50, SR, and SP. SP denotes the so-called split between the fishing power of the test and control trawls. Although it is of no real interest here, it is necessary to assess the value of SP to obtain the value of the selection parameters L50 and SR (see Wileman et al. [23] for further information on this subject). Function (3) is written as:
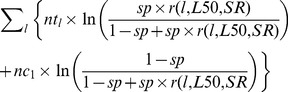
(3)where the summation is over length classes l. nt_l_ denotes the number of individuals found in the test gear (15.4 mm trawl) of length l, whereas nc_l_ denotes the number found in the control gear (7 mm trawl). Data were pooled for the four pairs of hauls prior to conducting the analyses to obtain the average size selection estimation for the 15.4 mm trawl.

Based on the estimated selection parameters L50 and SR, the length Li by which the retention likelihood is in % can be calculated by:
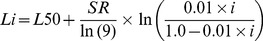
(4)Estimation of L05 to L95 based on (4) is required for the comparison between experimental- and simulation-based results by means of the method described in Herrmann et al. [13].

Estimation of 95% confidence intervals for the selection parameters and the entire selection curve as well as for L05 to L95 is carried out using a bootstrapping method implemented in the SELNET software.

### Simulation of size selection of krill

Based on the experimentally obtained size selection results for the commercial trawl and using CS1 and CS2 optimally rotated with optimal attack angle, we can explain the experimental results assuming contributions of different mesh oa values. This kind of netting contact corresponds to what has been assumed when using FISHSELECT to investigate size selection for different species of fish. Using the observed oa range derived from underwater video recordings during commercial fishing, we explored, by combining the contribution of meshes with oa values of a slightly wider range than observed, the possibility to obtain a size selection curve similar to the experimentally obtained selectivity curve for the commercial netting. For this, we used simulated data for the specific commercial mesh size with oa values of 15, 20, 25, 30, 35, 40, 45 and 50° using the method described in Herrmann et al. [13]. We divided the L05 to L95 values from the experimental data into 5% steps and investigated whether it, by combining the same oa-value as observed on underwater recordings, was possible by simulation to obtain a similar size selection curve as experimentally observed. Finally, we used the estimated distribution of oa values together with the FISHSELECT results for other mesh sizes to predict the size selectivity of krill for trawls with these different mesh sizes following the procedure described in [13].

## Results

### Mesh size and shape description

The shape of the 15.4 mm meshes appeared relative stable during commercial fishing based on underwater observation (Fig. 4, Table 2). Pictures from the underwater video were captured and five individual meshes were identified and digitized (Table 2). Table 2 also provides the model fit statistics for a diamond mesh description of the commercial netting. Based on its high R^2^ value, the meshes subsequently were described using the diamond mesh description. The five meshes listed in Table 2 covered the oa range in the captured picture frames, and the most open and closed meshes were selected for analysis. Table 2 indicates that 25–45° is the realistic oa range during commercial fishing for krill in the tapered part of the trawl design used in this study.

**Table 2:** Mesh size measurements based on image analysis (mesh id 1–5) and fit statistics for using a diamond mesh description (R^2^) and measured mesh opening angles (oa values in degrees) based on underwater video recordings during commercial fishing (mesh id 6–10).

Mesh id	Mesh size (mm)	Mesh id	R2	oa (degrees)
1	15.08	6	0.9511	44
2	15.4	7	0.9881	34
3	15.81	8	0.9167	28
4	15.94	9	0.8709	31
5	14.66	10	0.9762	33
**Mean**	**15.38**		**0.9406**	**34**

### Use of preserved krill for morphological measurements

No significant difference in morphology was found between fresh krill collected from the trawl catch and those stored in borax-buffered 4% formalin for 10 months (total body length: F = 0.1145, P = 0.9; carapace width: F =  0.1266, P = 0.9). The minor differences in these parameters (Table 3) likely are due to slight differences in the locations at which measurements were taken and in interpretation of the caliper readings.

**Table 3:** Dates, body length and carapax width measurements of 30 juvenile, sub adult and adult krill collected fresh from the trawl catch (25. January) off South Orkney Islands and after two and 10 months preserved in borax-buffered formalin (4%).

Date in 2012	Range body length (mm)	Mean (SD) body length (mm)	Range body width (mm)	Mean (SD) body width (mm)
25. January	27.0–55.0	44.7 (7.7)	2.4–7.1	4.7 (1.1)
21. March	27.0–55.0	44.3 (7.6)	2.2–7.0	4.7 (1.1)
14. November	27.0–54.0	43.8 (7.4)	2.5–7.2	4.8 (1.1)

### Morphological measures and penetration models

The three measured cross sections had different cross section shapes. CS1 was best described by a flexelipse1, CS2 was best described by a flexelipse3, and CS3 was best described by a flexdrope2 (Table 4, Appendix S1). These models were chosen for use in the subsequent analyses because they displayed the highest R^2^ value and the lowest AIC value. Figure 2 shows both the actual and the modeled cross section shapes and that they were in agreement. The model description of CS2 (lower part) did not include parts of the legs that were present in the cutting zone (Fig. 2). Krill legs are expected to have little effect on the optimal orientation, as they should fold up against the ventral side of the animal during mesh penetration with optimal orientation (head or tail first). Table 5 shows the length-based regression parameters for body length versus width and height in the cross sections.

**Table 4:** Fit statistic for the used models to describe CS1, CS2 and CS3.

Cross section (CS)	Model	R2	AIC
1	flexellipse_1	0.8334	−53.63
2	flexellipse_3	0.7427	−37.32
3	flexdrope_2	0.8670	77.97

The model description for each cross section is based the highest R^2^ value and the lowest AIC value among the tested models (see appendix S1).

**Table 5:** Estimated regression coefficients for CS1 and CS2.

	CS1		CS2	
	Length vs. width	Length vs. height	Length vs. width	Length vs. height
a	0.1031	0.1444	0.0995	0.1282
b	0.0033	0.7278	0.0833	0.2461
R2	0.8478	0.8220	0.8822	0.8419

### Comparison of FISHSELECT-based and experimental selectivity estimates

**Figure 6:**
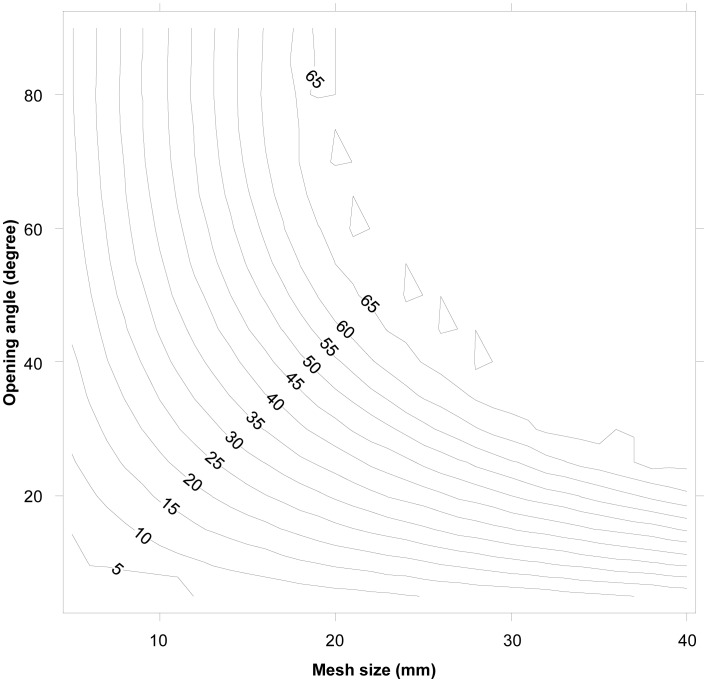
Diamond mesh design guide for Krill, based in combination of CS1 and CS2. The plot gives iso-L50 curves as a function of mesh size (mm) and mesh opening angle (oa).

The design guide based on CS1_CS2 was used to predict the basic selective properties for krill for all relevant sizes of diamond mesh when the individuals meet the meshes at the optimal orientation (Fig. 6). The design guide covers the mesh size range from 5 to 40 mm and the opening angle range from 10 to 90°. The size selectivity of krill depends greatly on the mesh opening angles. This is especially true for meshes with oa values ranging from 10–45° where meshes with larger oa values have less effect on the predicted L50. The predicted L50 for a given mesh size is however increasing towards a mesh opening angle of 90° (which equals a square mesh). The design guide further indicates that even the small meshes used in some survey trawls (∼7 mm) can be selective if the meshes are sufficiently open; if true, such surveys may underestimate the density of juvenile krill.

**Figure 7:**
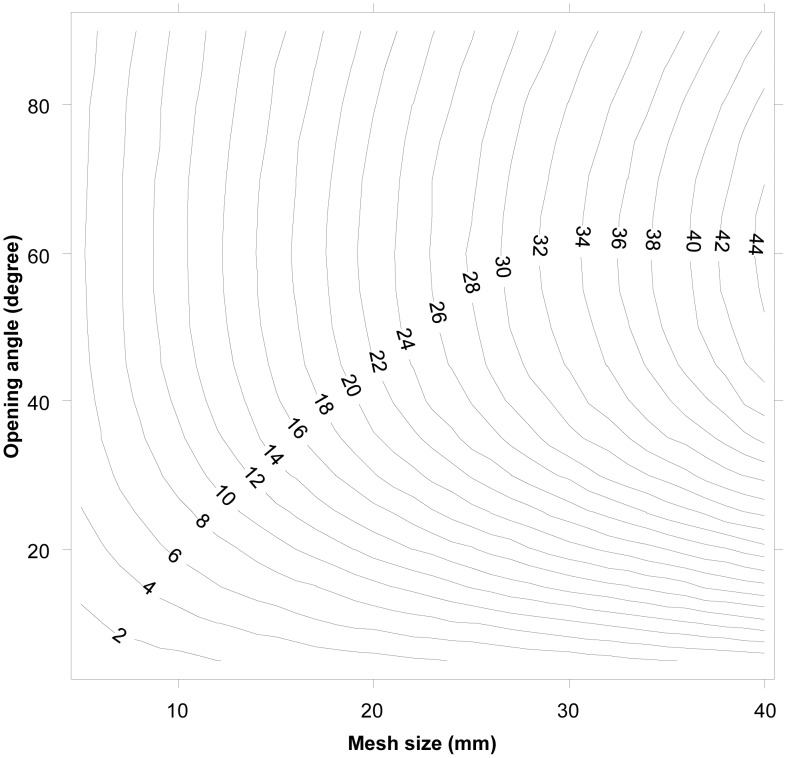
Diamond mesh design guide for CS3. The plot gives iso-L50 curves as a function of mesh size (mm) and mesh opening angle (oa).

**Figure 8:**
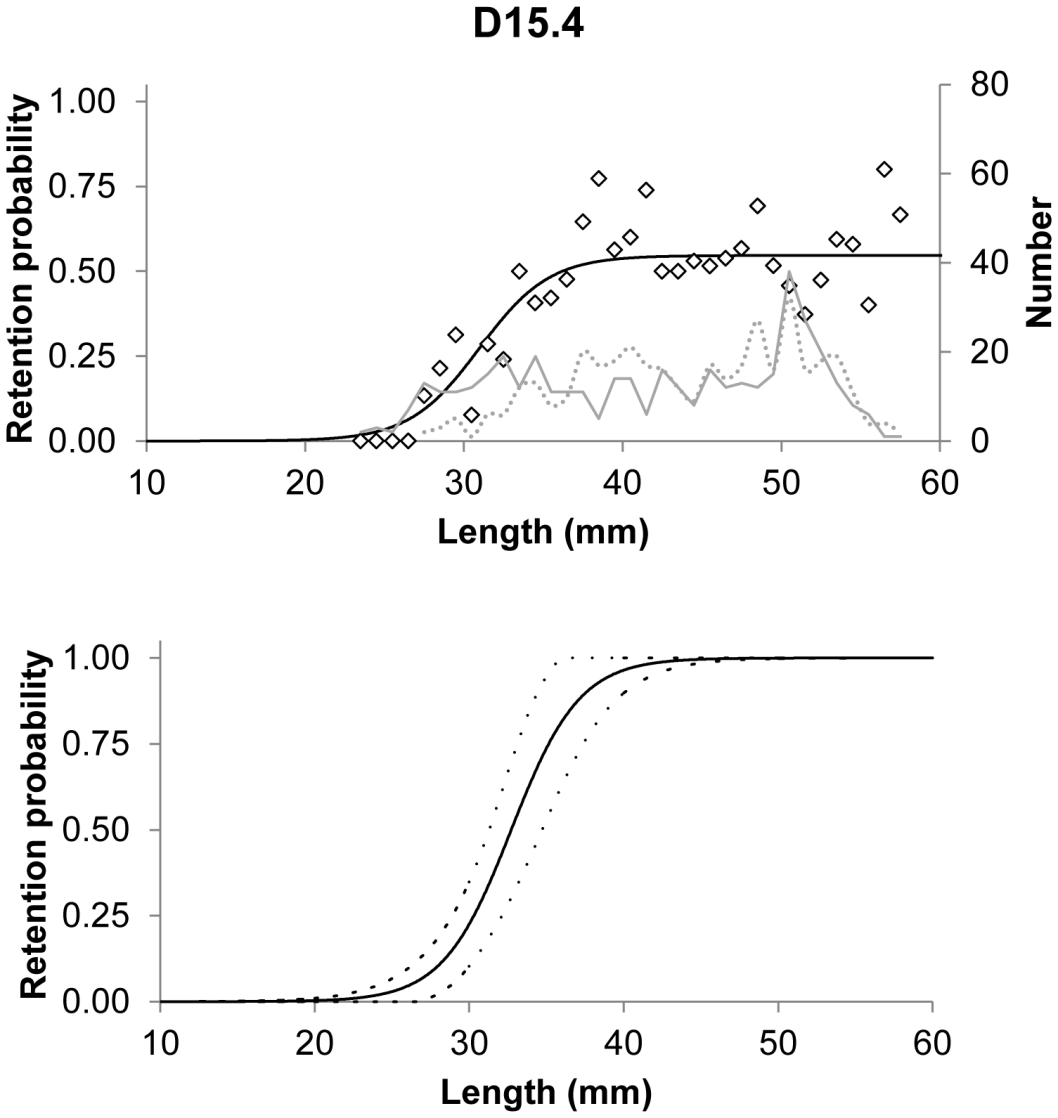
Top plot show paired gear fit of the experimental data. The population structure of the measured individuals are indicated (solid line = 7 mm survey trawl (*Macroplankton trawl*); broken line 15.4 mm commercial trawl). The lower plot is the size selection curve including 95% confidence limits.

**Figure 9:**
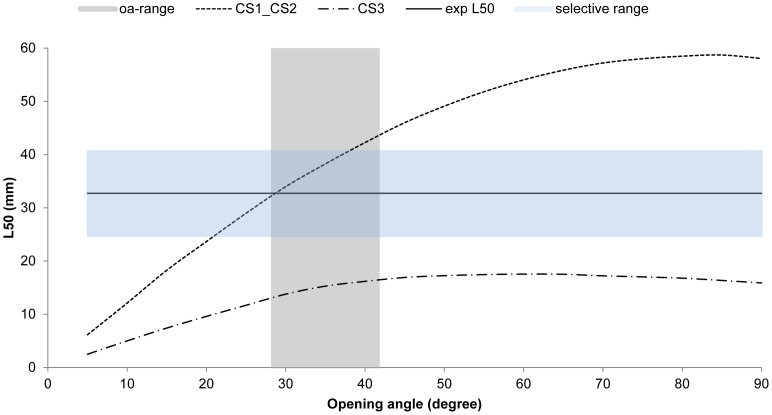
The experimentally obtained L50 value is indicated with the solid line (exp L50). L50 predictions for CS1_CS2 and for CS3 are indicated for the commercial mesh size (15.4 mm). The realistic mesh opening angles during commercial fishing is indicated with the vertical gray interval. The horizontal interval indicated the selective range for the 15.4 mm commercial mesh size.

Compared to the contact mode CS1_CS2, little escapement occurs for the larger CS3 cross section, even for rather large meshes (Fig. 7). When the meshes of the commercially used trawl (15.4 mm) are open optimally, krill smaller than 18 mm length can potentially escape (Fig. 7). The experimentally obtained results show that L5 (5% retention likelihood) is above 26 mm (Table 6), which means that this type of contact plays no role in defining the size selection for krill in this type of gear. In reality, we retained only 50% of the individuals with a body length of 33 mm (L50) (Table 6). The experimental L50 of krill with the 15.4 mm commercial trawl was estimated to be 32.72 mm with an SR (L75–L25) value of 4.85 mm (Table 7). The selectivity curve shown in Figure 8 demonstrates that size selectivity occurs for individuals smaller than 40 mm in the commercial trawl. Based on the fit statistics in Table 7 it is demonstrated that the applied model (2) in Fig. 8 is able to describe the experimental data sufficiently (p-value>0.05). The experimental selectivity results also show that fewer than 5% of the krill smaller than 26 mm length that enters the trawl will be retained (Table 6). Based on this result, any contribution to the size selectivity of krill from contact modes with L50 less than 26 mm will be very limited. Furthermore, the majority of individuals seem to be able to meet the meshes with a far more optimal body orientation (CS1_C2), at least for their decisive (last) contact with the netting. This is clarified in Figure 9, in which the potential selectivity based on CS1_CS2 and CS3 is compared to the observed selective range in the commercial netting (15.4 mm), the observed oa range during commercial fishing, and the experimental L50 value. Selectivity based on CS3 does not reach the experimentally observed selective range; in contrast, the estimated selectivity based on CS1_CS2 has reasonable overlap with both the expected experimental oa range and the selective range for the 15.4 mm mesh size (Fig. 9). However, Figure 9 shows that results could be slightly biased towards smaller oa values. Such difference may result from effect of attack angle with netting or none optimal rotation for krill during contact with the netting. This potential effect is investigated in detail in the next section.

**Table 6:** Values for the length of krill (L5-95) with different fixed retention likelihoods based on selectivity data from experimental fishing.

L	Value (mm)
5	26.23 (23.80–28.54)
10	27.87 (25.80–29.83)
15	28.90 (27.04–30.59)
20	29.66 (27.92–31.24)
25	30.30 (28.69–31.79)
30	30.85 (29.33–32.27)
35	31.36 (29.84–32.74)
40	31.83 (30.27–33.19)
45	32.28 (30.71–33.68)
50	32.72 (31.09–34.12)
55	33.16 (31.53–34.58)
60	33.62 (31.92–35.10)
65	34.09 (32.32–35.64)
70	34.59 (32.73–36.28)
75	35.15 (33.20–36.93)
80	35.78 (33.66–37.78)
85	36.55 (34.13–38.75)
90	37.57 (34.96–40.03)
95	39.22 (36.19–42.21)

95% confidence limits in indicated in brackets.

**Table 7:** Selectivity estimates for the commercial 15.4-statistics based on experimental fishing.

L50 (mm)	32.72 (±1.74)
SR (mm)	4.85 (±2.10)
SP	0.55 (±0.04)
p-value	0.26
Deviance	36.80
DF	32
Total number in test	393
Total number in control	416

95% confidence limits in indicated in brackets.

### Effect of cross section orientation and attack angle

**Figure 10:**
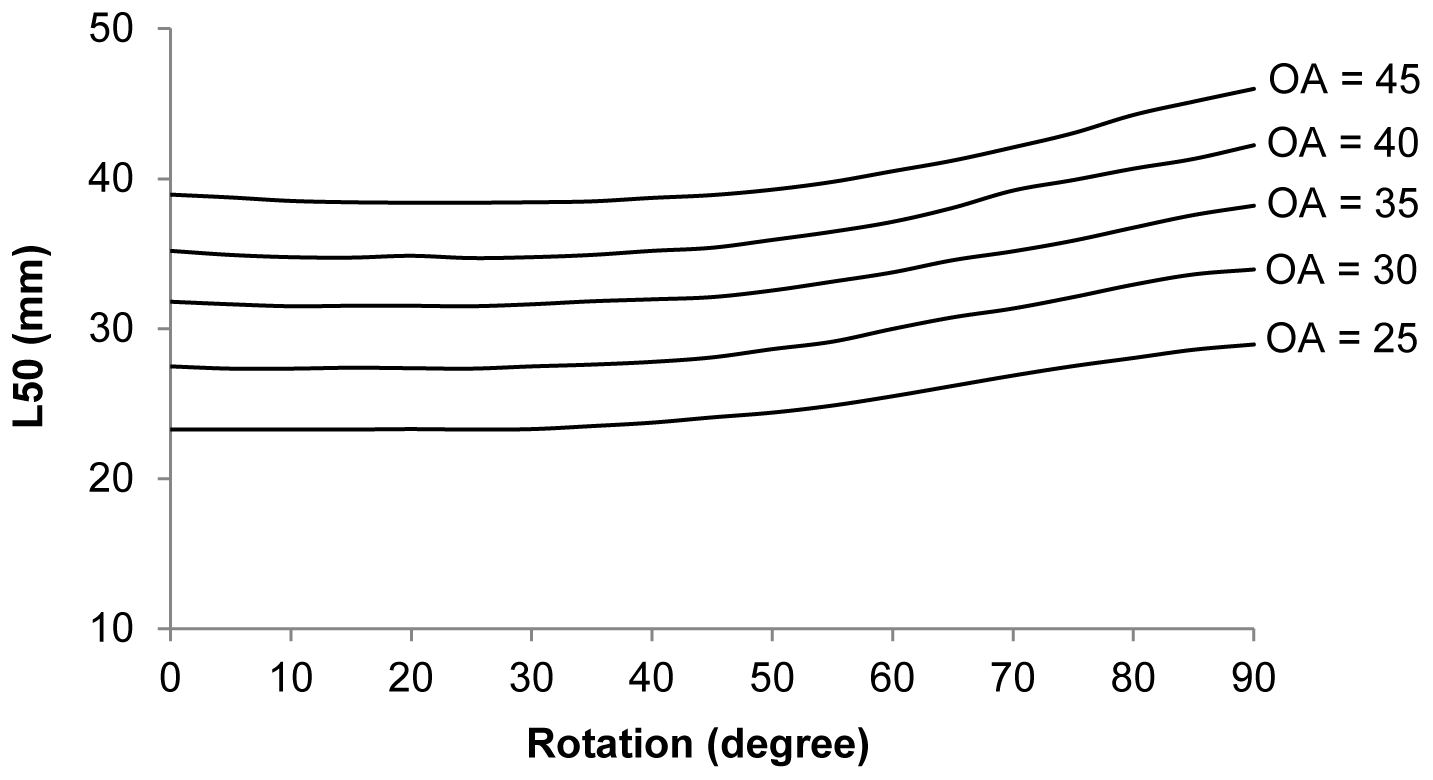
The effect of krill encountering meshes with different orientation. The penetration model (CS1_CS2) is rotated, at optimal attack angle perpendicular to the netting from 0–90°. 0° is a dorso ventral orientation equal to normal swimming orientation.

When examining the effect of cross section orientation and attack angle, we used only CS1_CS2, as CS3 was found to have no effect on the size selection of krill in the current trawl design. Figure 10 shows the potential effect of orientation of CS1_CS2 in the mesh for the relevant oa range rotated from a dorso-ventral orientation (see CS1 and CS2 in Fig. 2) (i.e., 0 to 90°). For the rotation range from 0 to 40°, the L50 value was nearly constant, indicating little effect of orientation over this range; it was only about 15–20% smaller than the maximal value obtained at 90° (Fig. 10). This relatively limited effect of rotation is also visible in Figure 5, but shows that individuals rotated 70–90° contrary to the other orientations will be retained by the mesh. Overall, the effect of cross section orientation in the mesh opening is relatively weak for a large range of rotation angles. This is due to the cross section shape of CS1_CS2, which is reasonably round shaped.

**Figure 11:**
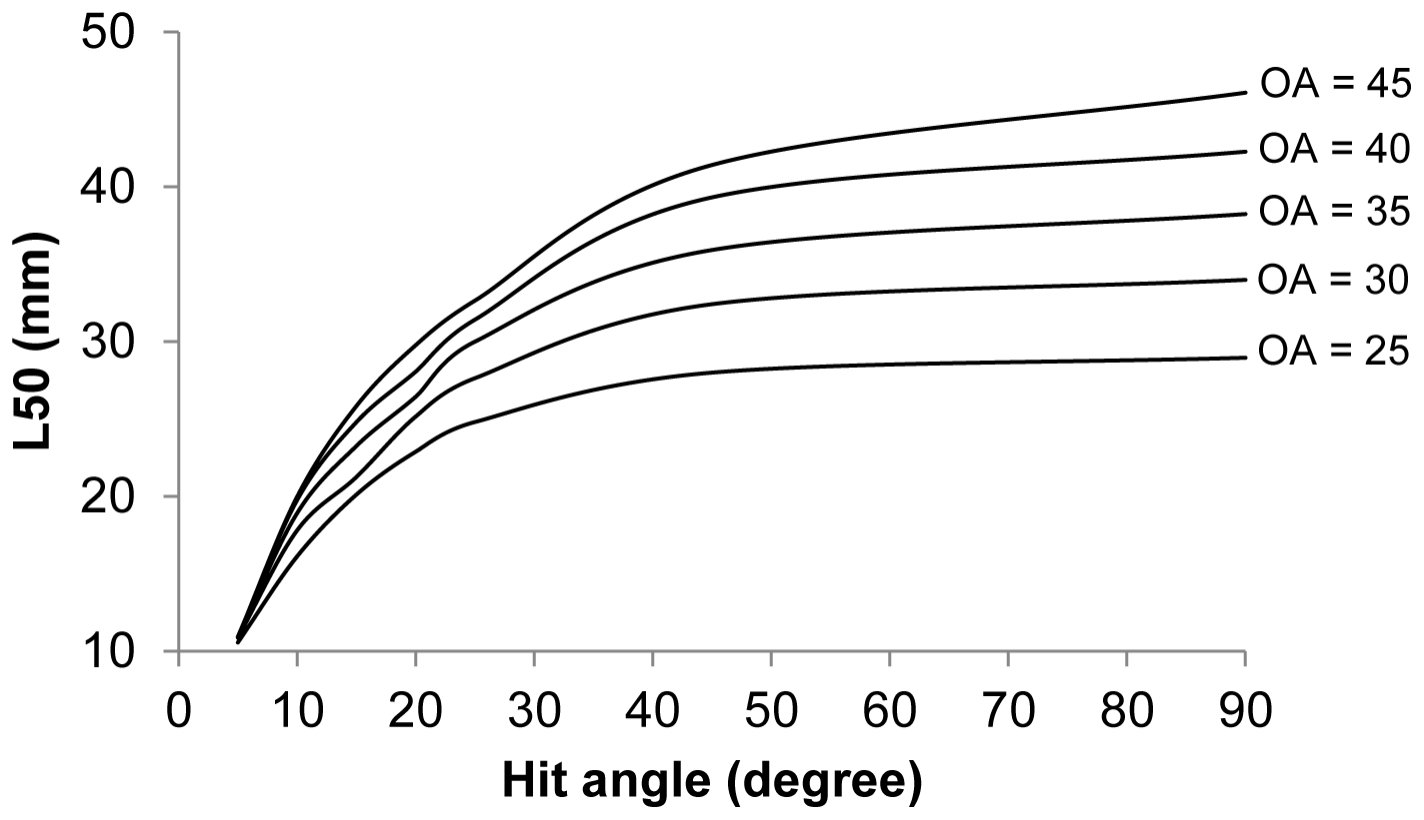
The effect of varying attack angles in the penetration model for CS1-CS2 in the range of oa-values that were found relevant in the 15.4 mm trawl.

A more dramatic effect on the estimated L50 value was predicted for low attack angles (0–30°) but little effect was detected for large attack angles (Fig. 11). The very low tapering present in the commercial trawls targeting krill results in attack angles <5°. If the angle of attack had an important effect on the size selection of krill in the trawl designs tested, we would expect L50 values of around 10 mm for the experimental results, which was not is the case (Fig. 8).

**Figure 12:**
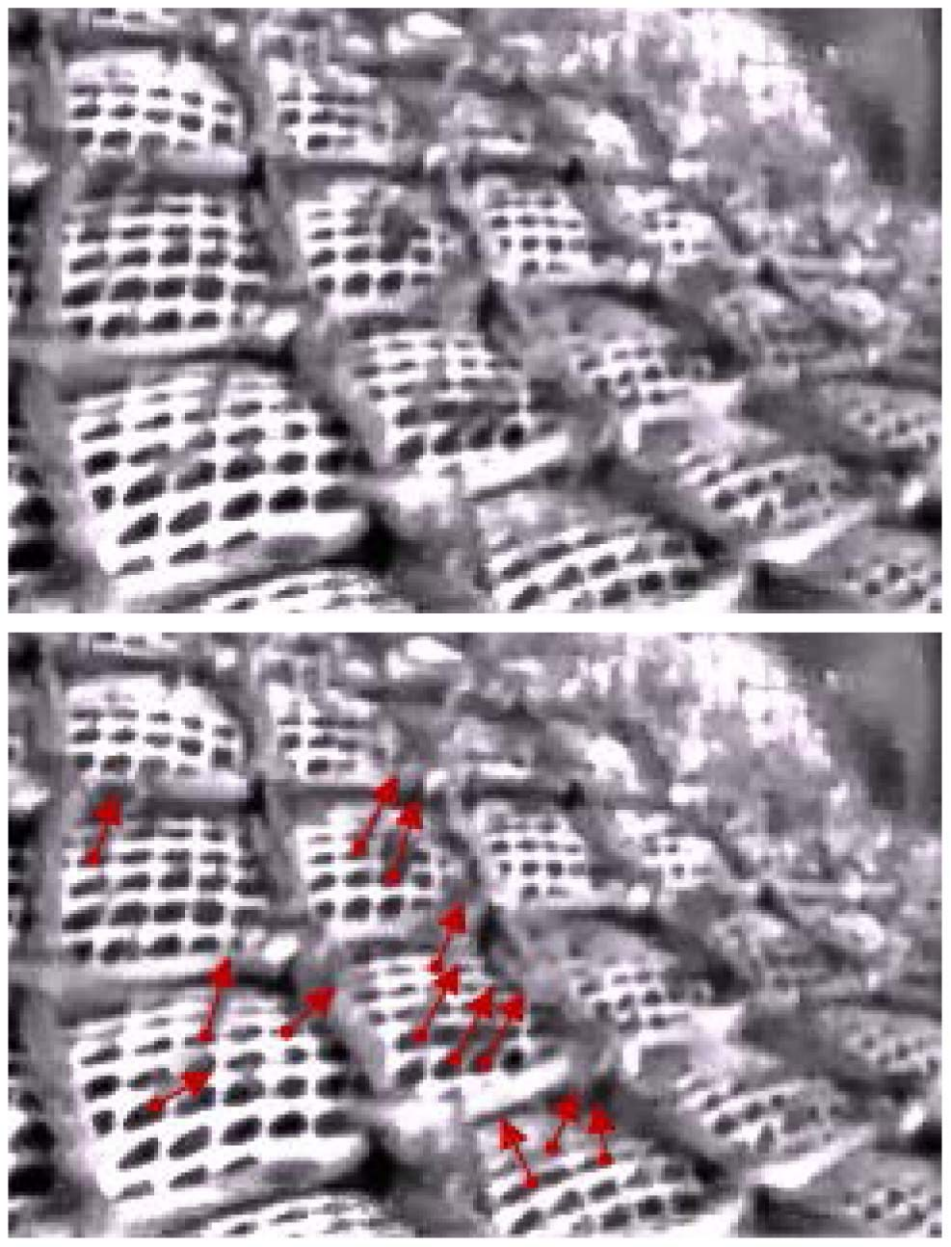
Underwater images captured during fishing indicating escaping krill in the 15.4 The escapees are marked with red arrows in the lower photo and clearly demonstrate an optimal orientation of the krill escaping. The photo is taken 10 meters in front of the cod line during commercial fishing.

The underwater recordings of escaping krill seem to indicate that they escape at the optimal attack angle perpendicular to the netting and head first (Fig. 12). This result demonstrates that the optimal FISHSELECT mode (i.e., CS1_CS2 with optimal orientation and attack angle) is a good approximation of the escape process. We therefore based our predictions of size selectivity of krill in trawls with other mesh sizes on this approximation and used only the optimal orientation (CS1_CS2).

### Simulation-based predictions and comparison to sea trail results

**Figure 13:**
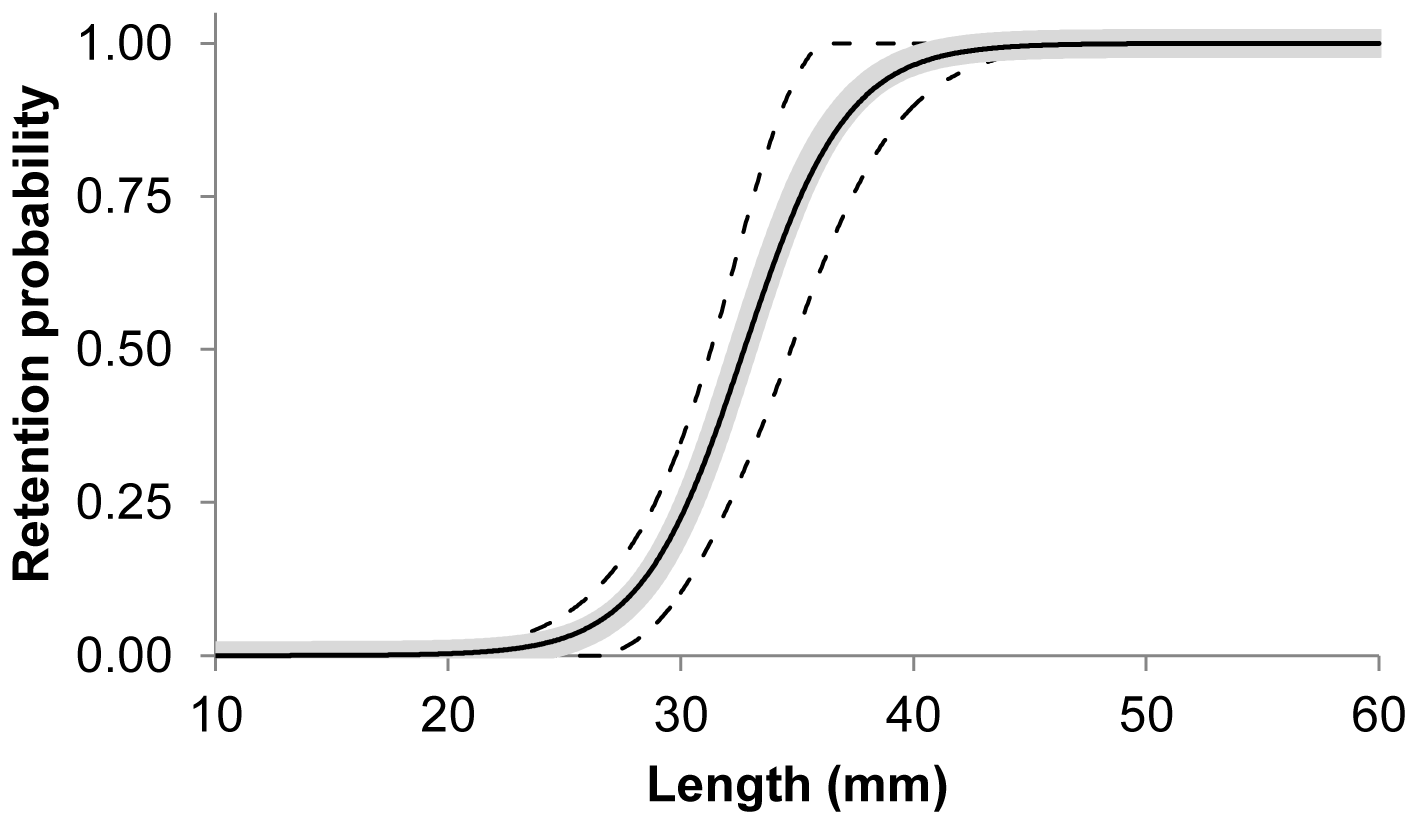
Experimentally obtained data (black line) with 95% confidence limits (broken line). Thick line (gray) is the predicted selectivity curve based on morphological based measurements (FISHSELECT) and the distribution of opening angle (oa)-values given in table 8.

**Figure 14:**
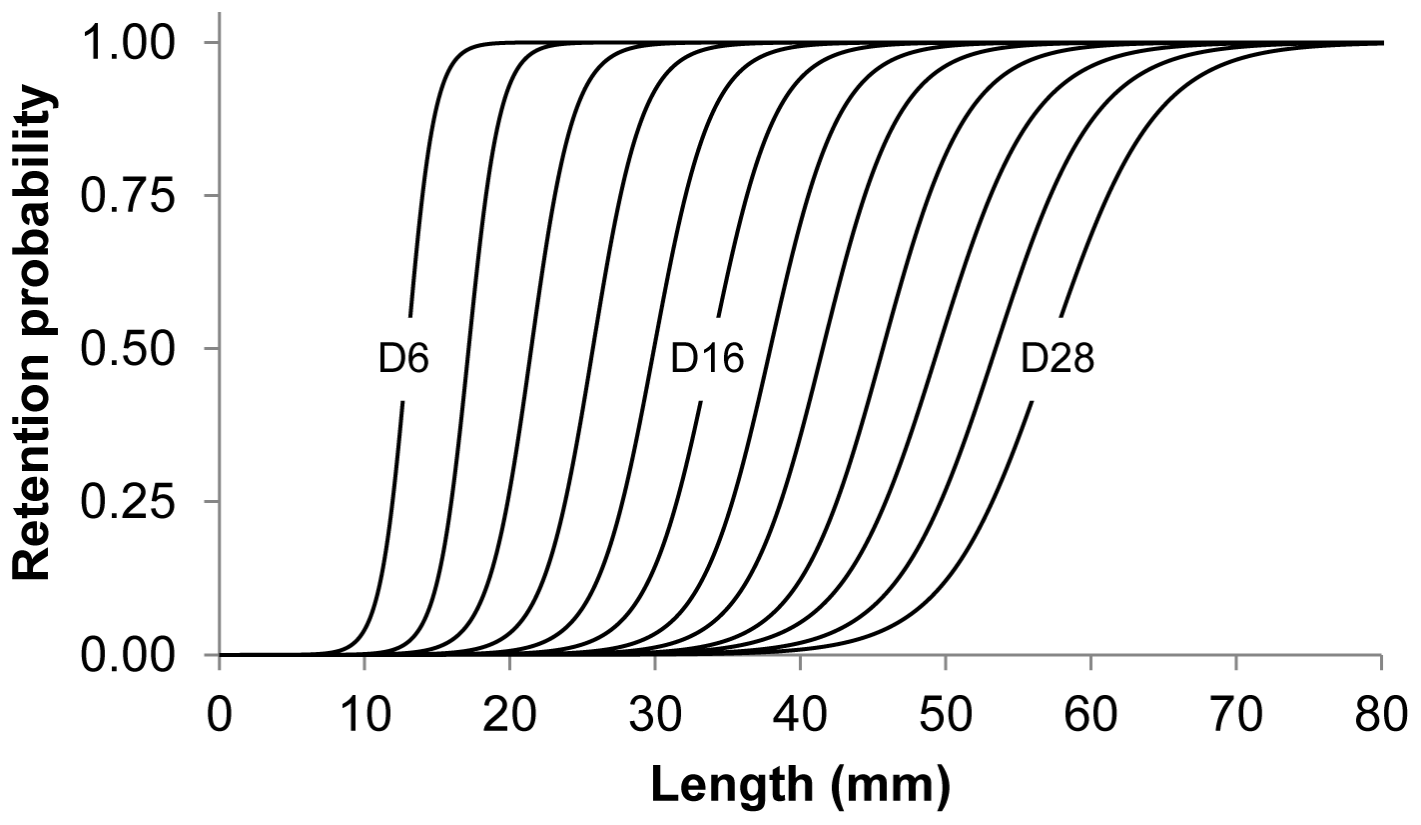
Predicted selectivity of krill in different mesh sizes based on the weight factors of the different opening angle (oa)-values for optimal CS1_CS2. Predictions are made from 6 to 28

In Figure 13, the experimentally obtained selectivity results are indicated by the black curve, and the dashed lines show the 95% confidence limits. The thick grey curve shows the optimal FISHSELECT-based predictions for CS1_CS2 at optimal orientation and attack angle using the relative contributions of oa values according to Table 8. The similarity of the two curves indicates that it is possible to obtain a modeled size selection curve that is very similar to the one obtained experimentally by using realistic oa values. Table 8 also shows that this curve is reproduced nearly exclusively by contributions of meshes with oa values of 25, 30, and 35°, with contributions of 39.9, 45.33, and 14.74%, respectively. These results also indicate that less open meshes are more common than what would be expected based on the underwater recordings. This might be due to the effect of non-optimal rotation and/or the effect of attack angle. However, the size selection for krill seems to be well approximated by the FISHSELECT optimal mode, as was previously found to be the case for a number of fish species. Thus, it makes sense to make predictions based on the FISHSELECT optimal mode. This premise is validated by the similarity between the experimental and predicted selectivity curves shown in Figure 13. Figure 14 and Table 9 shows the predictions of size selectivity for krill using the oa distribution in Table 8 for the optimal orientation of CS1_CS2 for the range of mesh sizes from 6 to 28 mm. This figure shows the size selectivity consequences of using different mesh sizes in the krill trawl fishery, and it is valid under the assumption that trawls with these mesh sizes have a similar distribution of oas during fishing.

**Table 8:** Simulated distribution of opening angles (oa) grouped in 5 cm. intervals that will result in identical selectivity curves for krill between simulated and experimental data.

oa (degree)	Contribution (0–100%)
15	0.00
20	0.01
25	39.90
30	45.33
35	14.74
40	0.01
45	0.01
50	0.00

**Table 9:** Predicted selectivity parameters (L50 and SR) for diamond mesh size ranging from 6 mm to 28 mm.

Mesh size (mm)	L50 (mm)	SR (mm)
6	12.96	2.06
8	17.17	2.39
10	21.48	3.25
12	25.72	3.81
14	29.93	4.34
16	33.91	4.83
18	37.93	5.24
20	41.55	5.77
22	45.68	6.35
24	49.49	7.22
26	53.44	7.56
28	57.20	7.97

## Discussion

We identified, measured, and parameterized the morphology that determines size selectivity of krill in towed gears by applying the FISHSELECT method with the modifications necessary for analyzing krill. Using the combination of this morphological description, realistic oa values for the meshes based on underwater observations made during commercial fishing operations, and experimental selectivity data, we were able to predict the selectivity for krill for different mesh sizes. This is the first time this has been attempted for krill, and it will be a useful tool for predicting the size selectivity of existing netting configurations and optimizing the size selectivity of future trawls designs based on management specifications in relation to the size selection. Such a predictive tool is especially valuable in expanding fisheries, such as the Antarctic trawl fishery for which few experimental data are available.

We expected to find more random selectivity for krill than that generally observed for fish in trawls due to the relatively high towing speed used to fish for these small individuals. The tapering in commercial krill trawls is very low compared to that of traditional fish trawls, which results in a low attack angle relative to the flow direction during towing. Theoretically, this could dramatically reduce the estimated L50 value. We found that the selectivity of krill in the commercial trawl can be explained by assuming that individuals of all lengths meet the meshes in the optimal contact mode (CS1_CS2) at a more or less optimal attack angle. Underwater recordings made during commercial trawling show that krill escape the trawl head first and relatively perpendicular to the netting wall. This suggests that individual krill are capable of orientating themselves in relation to the trawl netting and meeting the meshes at an optimal orientation and attack angle. An alternative explanation for the observed size selectivity of krill is that selection is a more random process but the size of the commercial trawl (about 200 m long) provides so many contacts with the netting during passage to the codend that the catch loses the signature of a random process, as most individuals meet the mesh opening optimally at some point during transport through the trawl. Further studies are needed to determine which of these two processes is actually occurring.

We estimated an L50 of 32.72 mm, which indicates that there is substantial size selectivity in the fisheries that use the 15.4 mm mesh size. The SR value for krill is small compared to the experimentally observed SR values for other crustaceans such as *Nephrops* [14]. Possible explanations for the difference are that krill do not have claws and that the length of *Nephrops* trawls typically are much shorter than e.g. krill trawl which reduces the number of netting contacts during the catching process. The observed stability of the mesh opening (oa values) in the krill trawl during fishing may also result in a lower SR value.

Because a relatively large proportion of the length classes of krill (24–42 mm) potentially can escape through the commonly used mesh size, it is important to estimate the survival of escapees in such fishing gears. Siegel [5] estimated mortality rates of 5–25% of krill individuals escaping through the trawl netting. This estimate was based on the assumption that the mortality rate of the individuals passing through the net meshes equals the rate of lethally damaged individuals observed in the codend of the commercial trawl. If this is correct, the total mortality caused by the commercial fishery might be considerably higher than catch values that are reported to the CCAMLR. However, several substantially different trawl designs, using different mesh sizes, are used in the commercial krill fishery. The potential mortality and survival rate of escapees likely depend on the different gear designs used. If the survival rate of escapees in krill trawls is low and differs between trawl designs, it is important to apply gears that are the most sustainable.

## Supporting Information

Appendix S1 (DOCX)
