# Supplementary material for: Size Selection of Antarctic Krill (Euphausia superba) in Trawls
Source: PLoS One. 2014 Aug 8;9(8):e102168. doi: 10.1371/journal.pone.0102168 (PMC4126659; doi:10.1371/journal.pone.0102168)
Supplement: Appendix S1 — (DOCX) [file pone.0102168.s001.docx]

**Appendix S1**

Describing the cross section shapes of krill in FISHSELECT requires a representation in polar coordinates (θ, r), where θ is the angle (0–360º) and r is the corresponding radius (see appendix in Herrmann et al. (2009)). A description that involves only a few parameters is preferred. One flexible method, which enables the modeling of a large family of different shapes using only a few parameters, is to use a parametric description in Cartesian coordinates of the following form (Bers and Karal 1976):

$\begin{matrix} x=f(t) \\ \\ y=g(t) \end{matrix}$ $\mathrm{t}\in\left[0,360\right]$

The actual shape is then defined by the selected formulas for the two functions f(t) and g(t).

The polar representation of the points on the cross section surface is then calculated by:

$\begin{matrix} r=\sqrt{x^{2}+y^{2}} \\ \theta=\tan^{-1}(\mathrm{y},\mathrm{x}) \end{matrix}$, where our representation returns the angle in the correct quadrant.

To represent the cross sections of krill, we needed to find mathematical descriptions for the two functions (f(t) and g(t)) with as few free parameters as possible but that still could describe the main characteristics of the cross section shapes of the species. During initial experimentation test of different new formulas based on trigonometric functions in the FISHSELECT software tool, we discovered that in addition to the ellipse, which is a standard and well-known shape with two parameters (c_1_, c_2_), several other descriptions with up to three parameters (c_1_, c_2_, c_3_) would potentially be able to produce points that together would generate shapes similar to those of the different cross sections of krill. For these shapes, the functions f(t) and g(t) are given by:

| Model | $f(t)$ | $\mathrm{g}(t)$ |
| --- | --- | --- |
| Circle | $c_{1}\times\cos\left(\pi\frac{t}{180}\right)$ | $c_{1}\times\sin\left(\pi\frac{t}{180}\right)$ |
| Ellipse | $c_{1}\times\cos\left(\pi\frac{t}{180}\right)$ | $c_{2}\times\sin\left(\pi\frac{t}{180}\right)$ |
| Bottle | $c_{1}\times\sin\left(\pi\frac{t}{180}\right)+c_{1}\times c_{3}\times\sin\left(\pi\frac{t}{90}\right)$ | $-c_{2}\times\cos\left(\pi\frac{t}{180}\right)+c_{1}\times c_{3}\times\cos\left(\pi\frac{t}{45}\right)$ |
| Flex_1 | $c_{1}\times\sin\left(\pi\frac{t}{180}\right)+c_{1}\times c_{3}\times\sin\left(\pi\frac{t}{30}\right)$ | $c_{2}\times\cos\left(\pi\frac{t}{180}\right)+c_{2}\times c_{3}\times\cos\left(\pi\frac{t}{90}\right)$ |
| Flex_drope_2 | $c_{1}\times\sin\left(\pi\frac{t}{180}\right)+c_{1}\times c_{3}\times\sin\left(\pi\frac{t}{90}\right)$ | $-c_{2}\times\cos\left(\pi\frac{t}{180}\right)$ |
| Flex_ellipse_1 | $c_{1}\times\sin\left(\pi\frac{t}{180}\right)$ | $-c_{2}\times\cos\left(\pi\frac{t}{180}\right)+c_{3}\times\cos\left(\pi\frac{t}{90}\right)$ |
| Flex_ellipse_3 | $c_{1}\times\sin\left(\pi\frac{t}{180}\right)-c_{1}\times c_{3}\times\sin\left(\pi\frac{t}{90}\right)$ | $-c_{2}\times\cos\left(\pi\frac{t}{180}\right)+c_{2}\times c_{3}\times\cos\left(\pi\frac{t}{90}\right)$ |
| Ship | $c_{1}\times\sin\left(\pi\frac{t}{180}\right)$ | $-c_{2}\times\cos\left(\pi\frac{t}{180}\right)+c_{3}\times\cos\left(\pi\frac{t}{45}\right)$ |
| Shoe_2 | $c_{1}\times\sin\left(\pi\frac{t}{180}\right)+0.1\times c_{1}\times\sin\left(\pi\frac{t}{45}\right)$ | $-c_{2}\times\cos\left(\pi\frac{t}{180}\right)$ |
| Soft_triangle | $_{1}\times\sin\left(\pi\frac{t}{180}\right)+c_{1}\times c_{3}\times\sin\left(\pi\frac{t}{90}\right)$ | $-c_{2}\times\cos\left(\pi\frac{t}{180}\right)+c_{2}\times c_{3}\times\cos\left(\pi\frac{t}{90}\right)$ |

Quantification of the ability of a particular shape to describe the experimentally collected data for a cross section of a fish can be based on calculation of the R^2^ value for the fit of the model to the data. The R^2^ value expresses the fraction of the variation in the data accounted for by the model within the total variation in the data. Using the polar expression (ϴ,r) for the points along the cross section shape, the R^2^ value for the shape fit can be calculated for each angle ϴ to compare the radius values *r* based on the model against based on the experimental data. The total variation in the data is calculated as the variance in the r values from the experimental data. Thus, while the R^2^ value can never exceed 1.0, a value close to 1.0 implies that the model describes the shape data well. Everything else being equal, the model with the highest R^2^ is preferable. However, a more flexible model requiring a larger number of parameters to define the shape would in general be expected to produce a higher R^2^ value. To assess whether the improvement gained in the modeling of the shape is worth the cost of the increased number of model parameters, the mean AIC value can be used to choose between competing models. The model with the lowest AIC value is the most preferable (Akaike 1973). We therefore used mean R^2^ values for the different shape models to evaluate their ability to describe the cross section shapes, and we used the AIC values to rank models with different numbers of parameters.
